# Progress of Microfluidic Continuous Separation Techniques for Micro-/Nanoscale Bioparticles

**DOI:** 10.3390/bios11110464

**Published:** 2021-11-18

**Authors:** Se-woon Choe, Bumjoo Kim, Minseok Kim

**Affiliations:** 1 Department of Medical IT Convergence Engineering, Kumoh National Institute of Technology, Gumi 39253, Korea; sewoon@kumoh.ac.kr; 2 Department of IT Convergence Engineering, Kumoh National Institute of Technology, Gumi 39253, Korea; 3 Department of Mechanical Engineering and Automotive Engineering, Kongju National University, Cheonan 1223-24, Korea; bumjoo@kongju.ac.kr; 4 Department of Future Convergence Engineering, Kongju National University, Cheonan 1223-24, Korea; 5 Department of Mechanical System Engineering, Kumoh National Institute of Technology, Gumi 39177, Korea; 6 Department of Aeronautics, Mechanical and Electronic Convergence Engineering, Kumoh National Institute of Technology, Gumi 39177, Korea

**Keywords:** microfluidics, separation, bioparticles, biosensors, biosample preparation

## Abstract

Separation of micro- and nano-sized biological particles, such as cells, proteins, and nucleotides, is at the heart of most biochemical sensing/analysis, including in vitro biosensing, diagnostics, drug development, proteomics, and genomics. However, most of the conventional particle separation techniques are based on membrane filtration techniques, whose efficiency is limited by membrane characteristics, such as pore size, porosity, surface charge density, or biocompatibility, which results in a reduction in the separation efficiency of bioparticles of various sizes and types. In addition, since other conventional separation methods, such as centrifugation, chromatography, and precipitation, are difficult to perform in a continuous manner, requiring multiple preparation steps with a relatively large minimum sample volume is necessary for stable bioprocessing. Recently, microfluidic engineering enables more efficient separation in a continuous flow with rapid processing of small volumes of rare biological samples, such as DNA, proteins, viruses, exosomes, and even cells. In this paper, we present a comprehensive review of the recent advances in microfluidic separation of micro-/nano-sized bioparticles by summarizing the physical principles behind the separation system and practical examples of biomedical applications.

## 1. Introduction

In recent decades, various microfluidic techniques have been developed to accurately control micro-/nanoscale bioparticles, such as trapping [1,2,3], focusing [4,5,6], compartmentalization [7,8,9,10], preconcentration [11,12,13], and separation [14,15,16] using mechanical, optical, magnetic, electrical, or chemical forces, resulting in improving the performance of biosensors [17,18,19]. Among the various particle manipulation techniques, separation of micro-/nanoscale bioparticles seems to be one of most essential processes for highly sensitive and selective biosensing and biochemical analysis of complex bio-samples, such as blood, which contains cells, bacteria, viruses, proteins, DNA molecules, and other biological ingredients. Therefore, the development of efficient and effective techniques for bioparticle separation leads to the development of innovative technologies and tools for biological sensing/diagnostics, as well as other biochemical processes in pharmaceutical, life sciences, and clinical analysis [20,21,22,23,24].

In general, the most widely used bioparticle separation process in conventional bioprocesses is a size-based centrifugation or filtration by porous membranes, which is easy to use due to its well-developed commercial equipment and consumables. However, the conventional method has an inevitable limitation in that it is difficult to separate particles in a continuous and autonomous manner as a batch process that operates periodically. More importantly, it seems to be unsuitable for separating bioparticles contained in a small amount of difficult-to-obtain biofluids or expensive/rare samples. On the other hand, microfluidic biosample separation technology can extract specific particles during continuous flow [6,11,25,26,27] and operate automatically for a long time [10,28,29,30,31]. Moreover, it is much easier to be developed as an all-in-one device that simultaneously performs sample preparation, including separation, preconcentration, and biosensing/bioanalysis, directly connecting the pretreated sample to downstream biosensors without additional processing [18,21,22,31,32,33].

**Figure 1:**
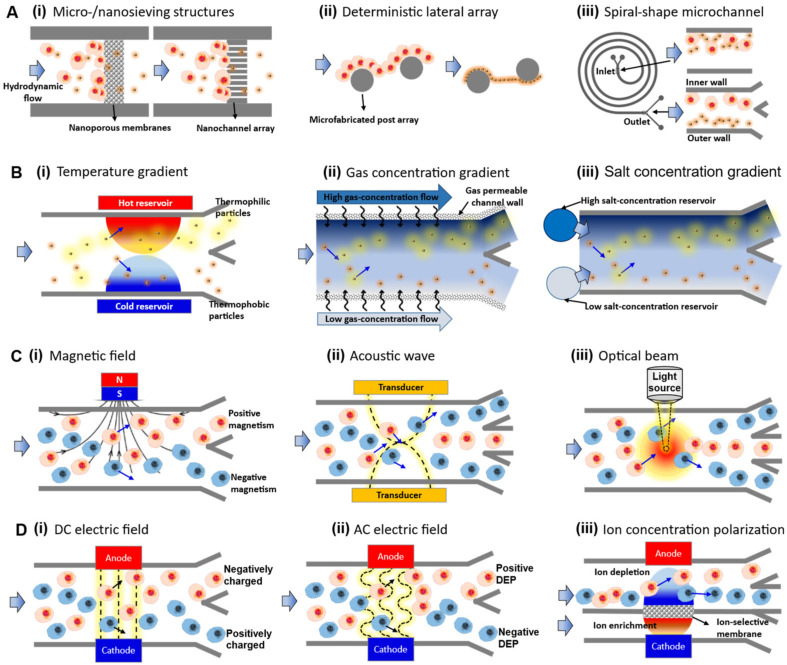
Schematic description of operational principles in microfluidic technologies for bioparticle separation. (**A**) Microfluidic passive separation group 1: hydrodynamic flow-based separation using (**i**) sieving micro-/nanostructures (filtration), (**ii**) deterministic lateral displacement arrays, and (**iii**) spiral-shape microchannels (inertial focusing); (**B**) Microfluidic passive-force separation group 2: microenvironmental gradients, such as (**i**) temperature gradient (thermophoresis), (**ii**) dissolved gas concentration gradient (diffusiophoresis), or (**iii**) electrolyte concentration gradient (diffusiophoresis); (**C**) Microfluidic active-force separation group 1: non-contacting mechanical force, such as (**i**) magnetic field (magnetophoresis), (**ii**) acoustic wave (acoustophoresis), and (**iii**) optical beam (optical tweezing); (**D**) Microfluidic active-force separation group 2: contacting electrical forces created by (**i**) DC electric field (electrophoresis), (**ii**) AC electric field (dielectrophoresis), or (**iii**) concentration polarization phenomena.

Here, we categorized various microfluidic separation techniques for micro-/nanoscale bioparticles into passive and active groups in this paper, depending on whether external equipment is needed to apply the momentum for particle separation. One group of passive separation methods can operate easily with simple experimental setups to only control hydrodynamic flow (passive separation group 1, Figure 1A). In group 1, once a hydrodynamic flow was introduced for delivery of a biological sample, size-based particle separation forces were spontaneously generated depending on the specific micro-/nanostructures, such as (i) sieving micro-/nanostructures (i.e., filtration), (ii) micropillar arrays (e.g., deterministic lateral displacement arrays), and (iii) specific shape-/cross-sectional profiles of microchannels (e.g., spiral-shape microchannels). This group had the advantage of being able to apply a relatively fast flow without using a separate active force, so it can be applied to various bioparticles, such as cells. However, in the case of separation of nanoscale bioparticles, such as DNA or proteins, there is a possibility that the fluid flow alone may not exert sufficient separation force.

Another passive separation method, in addition to hydrodynamic flow control, requires formation of microenvironmental gradients of basic experimental parameters, such as chemical concentration or temperature (passive separation group 2, Figure 1B). In passive group 2, a separation force was created using a gradient of chemical compositions or micro-environments of fluids inside the microchannel, such as (i) temperature gradient (thermophoresis), (ii) dissolved gas concentration gradient (i.e., diffusiophoresis), or (iii) electrolyte concentration gradient (i.e., diffusiophoresis). The micro-environment gradient used in this group had the advantage of tunable particle manipulation as it can be rapidly created/changed in a local spot of the microfluidic channel, but a spatiotemporally steep gradient (e.g., temperature) may limit the types of applicable bioparticles. Therefore, careful parameter setting according to the separation target was required.

By contrast, most active bioparticle manipulation methods that enable precise, selective, and on-demand migration controls utilize additional equipment/devices for applying external forces into microchannels, such as magnetic, acoustic, optical, or electrical forces. We classified microfluidic active separation techniques into active separation group 1 (Figure 1C) and group 2 (Figure 1D), according to whether force-generation components, such as magnets, transducers, beam sources, or electrodes, were in direct contact with sample fluids of microchannels. In active group 1, a particle separation force was applied by an external transducer or power source that was not in direct contact with the sample fluid. The active force, such as (i) magnetic field (i.e., magnetophoresis), (ii) acoustic wave (acoustophoresis), and (iii) optical beam (optical tweezing), was transmitted to the sample by penetrating the microchannel wall structures made of PDMS or glass. In the case of this active group, since the separation force did not come into direct contact with the biofluid, the characteristics of the material constituting the wall surface of the microchannel acted as a more important variable. For example, since it is difficult to apply a laser for optical separation through a microfluidic device made of an opaque material, it is necessary to select a material that has high transparency and does not generate well with heat or chemical reaction by reacting with light energy.

In group 2, a particle separation force was created by applying an external electrode set that was in direct contact with the electrolyte solution or sample fluid. The applied electric power generated (i) DC electric field (i.e., electrophoresis), (ii) AC electric field (i.e., dielectrophoresis), or (iii) concentration polarization phenomena for particle separation. In this group, it was important to maintain chemical homeostasis by continuously flushing the electrode channels connected to the sample channels, so the biproducts generated by chemical reactions and electrolysis occurring around the electrodes did not spread to the main isolation channel. At the same time, the separation power or Joule heating effects may be different depending on the concentration (conductivity) of the buffer solution, so it was useful for separating nanoscale particles with relatively high robustness, such as proteins and DNA, rather than separating sensitive bioparticles, such as cells.

In this paper, we reviewed recent research trends on microfluidic particle separation or extraction from complex biological samples, such as blood. Although many reviews on microfluidic particle separation have been reported so far [34,35,36,37,38,39], we believe that a new review that is specialized for biological particle separation and can provide a direction to overcome the bottleneck of the current technology is needed. Therefore, in this paper, we included the latest technologies, such as multiphysics, multiround, and massive parallelization techniques, that have the potential to solve the current issues [40,41,42,43]. Briefly, we first presented the physical, electrical, and biochemical properties of several bioparticles that are important for biosensing and biochemical in vitro diagnosis. Then, according to the driving force for separation, we examined the operational principle, pros and cons, scope of applications, and biocompatibility of the continuous microfluidic bioparticle separation technology. Lastly, we discussed current limitations in microfluidic bioparticle separation technologies and suggested future research directions by introducing several recent efforts to overcome the issues in microfluidic separation technology.

## 2. Characteristics of Bioparticles

### 2.1. Nucleotide Chain

Biological assays using deoxyribonucleic acid (DNA) and/or ribonucleic acid (RNA) are very important in the process of isolating/purifying nucleic acids from complex biosamples, such as cell lysates for high-sensitivity biosensing, such as forensic analysis [44] and disease diagnosis [45]. The diameter of DNA is approximately 2~3 nm, and the length varies depending on the size of the fragment, but it can be up to few meters long. Most of the DNA has a negative charge because of the negative charge of phosphate groups. The common methods of DNA separation are gel electrophoresis and spin column, which uses charge and size (length) of the samples [46]. In a typical column, tiny DNA moves very slowly due to small gravity, so an electric field is used in gel electrophoresis, in which DNA/RNA molecules are pushed by an electric field through hydrogels containing nanoporous structures. DNA travels through the pores of the gel at a rate that is inversely proportional to their length. This means that shorter DNA molecules travel longer distances through the gel than longer DNA molecules. Specifically, this field is applied so one end of the gel is positively charged and the other is negatively charged. Thus, the negatively charged DNA and RNA are pulled toward the positively charged end of the gel.

### 2.2. Protein

Proteins vary in size and shape at approximately 5~70 nm depending on the type [47]. The importance of proteins cannot be overemphasized because they provide structure, produce energy, and enable almost all basic biological phenomena, such as communication, movement, and reproduction through mass/ion transfer within cells [48]. In short, proteins provide a structural and functional framework for cell life. Most of the protein separation process is isolating a single type of protein from a complex mixture, such as serum, cell culture fluid, or tissue lysate, which is very important for medical diagnosis and disease detection. Protein purification is also essential to analyze its function, structure, and interactions. Separation of proteins, such as DNA, are difficult using only volumetric forces, such as gravity, due to their small size. However, most proteins also have different surface charge characteristics, so they can be separated by gel electrophoresis and isoelectric focusing, or chromatography under controlled electric field and pH [49].

### 2.3. Extracellular Vesicle (Exosome)

Exosomes are released by almost all cell types in the body, range in size from 30 to 150 nm, and are a type of extracellular vesicle containing essential cellular components, such as proteins and DNA/RNA. Exosomes are key mediators of cell-to-cell communication, carrying distinct cargoes of lipids, proteins, and nucleic acids that reflect the original cell [50]. Therefore, by analyzing exosomes secreted from cells that cause disease, such as cancer, it is possible to determine the biomedical status and diagnose the disease at the early and treatable stage. However, the separation of exosomes from other proteins and lipid particles represents a considerable challenge. The most widely applied method of separating exosomes is differential centrifugation [51]. The principle of this method is to separate exosomes from other matrix present in the sample according to their volume and physical properties.

### 2.4. Virus

Viruses are microscopic sources of infections that replicate inside all types of living cells, from animals and plants to microorganisms, and are the most diverse and abundant types of organisms. Most viruses studied are 20–300 nm in diameter, have lengths up to 1400 nm, and vary in size, shape, and surface charge [52]. The detection of viral particles is practically very important because virus infection remains one of the global issues, such as the COVID-19 pandemic. Various approaches are used to detect and identify viruses, such as polymerase chain reaction, microbial and biochemical tests, genetic engineering methods, and immunological methods [53,54], and to this end, selective separation/extraction of viruses from various biofluids is a key step. The major technical challenge with virus separation is the extraction of viruses from biosample matrices, such as blood, soil, sputum, etc., and it is common to rely on porous membranes, centrifuges, or chromatographic purification.

### 2.5. Bacterial Cells

Since all bacterial cells are prokaryotes, they do not have a nuclear membrane, and most bacterial cells are 1/10 the size of eukaryotic cells and are generally 0.2–5.0 μm [55]. Bacteria play a very important role in the balance and self-purification of the ecosystem, but disease-causing pathogens also exist. Thus, early diagnosis of bacteria is closely related to healthy human life, including drug discovery, clinical diagnosis, and food and water safety. Typical physical separation methods are centrifugation and filtration, but their efficiency is poor when the sample matrix is complex, such as blood and food. Other separation methods utilize surface charge density, hydrophobic/hydrophilic property, and specific binding affinity of bacterial cell membranes, such as antigen/antibody reactions [56].

### 2.6. Blood Cells

Blood is mainly composed of plasma, red blood cells (RBCs), white blood cells (WBCs), and platelets. The sizes of RBCs and WBCs are 5–8 µm and 10–20 µm in diameter, respectively. The main role of RBC is to transport oxygen from the lungs to the body tissues and to deliver carbon dioxide back to the lungs, and WBC plays a very important role in maintaining homeostasis in the immune system of the body against bacterial, viral, fungal, and parasitic infections. It is not difficult to separate plasma and blood cells by fractionating whole blood, but isolating specific cells from different types of leukocytes is a rather complex protocol using differences in cell size/shape or specific binding affinity. Particularly, it is difficult to separate in cases where blood contains very low concentrations of target cells, such as circulating tumor cells, because of the complexity of samples that include numerous types of cells and biomolecules [57].

## 3. Passive Separation Group 1: Hydrodynamics-Based Separation

### 3.1. Sieving/Mechanical Filtration

**Figure 2:**
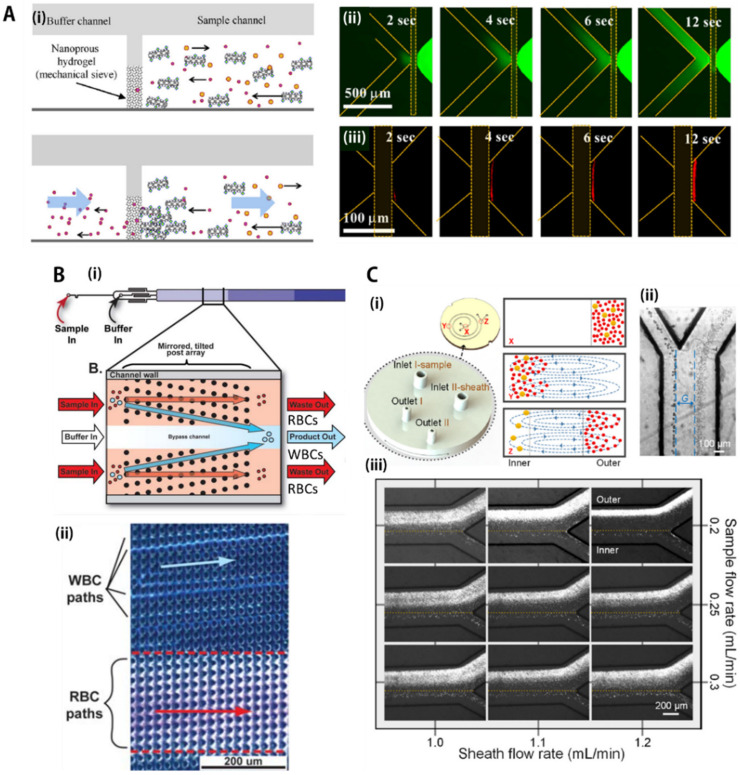
Microfluidic passive-force separation group 1: hydrodynamic, flow-based separation. (**A**) Size-based particle filtration using nanoporous hydrogel membranes as a sieving structure within microchannels. (**i**) Schematic description for showing the operational principle of the size-based filtration. (**ii**,**iii**) Experimental results of the separation. (**ii**) Non-target proteins with green fluorescence (**ii**) penetrated the hydrogel membrane, (**iii**) while red-fluorescent target biomolecules bound to long-molecular carriers (microtubules, >10 μm in length) were selectively filtered by the nanoporous membrane. Reprinted with permission from Elsevier B.V. [62]; (**B**) (**i**) Separation principle of deterministic lateral displacement (DLD)-based blood cell separation. (**ii**) Experimental results of blood separation using DLD device. Relatively large white blood cells moved to buffer channel, while smaller cells, such as red blood cells and platelets, remained in sample channel. Reprinted with permission from John Wiley & Sons, Inc. [65]; (**C**) (**i**) Operational principle of inertial separation in the syringe i-sorter. (**ii**) The separation of 7 μm and 20 μm particles at the outlet at the optimal flow rates of 0.2 mL/min and 1.2 mL/min for the sample flow and sheath flow, respectively; (**iii**) Distributions of tumor and blood cells at the outlet at varied flow rates. At all the tested flow rates, the tumor cells were focused near the inner channel wall (below the yellow dotted line), while the blood cells formed a relatively large band with varied widths near the outer channel wall. Reprinted with permission from Elsevier B.V. [66]. All rights reserved.

Microfluidic sieving is a method of separating particles according to their characteristic size by applying a flow to an integrated porous partition (e.g., membrane, hydrogel, or nanoparticle clusters), and the behaviors are dependent according to filter characteristics (e.g., pore size, pore density, and stiffness) and the direction and type of applied forces [58,59,60,61,62,63]. As shown in Figure 1A, Kim et al. [62] fabricated multilayered microchannel networks to integrate nanoporous hydrogels (~100 nm pore size) within microfluidic channel networks as mechanical sieving structures. As shown in Figure 2A(i), in the sample channel, the red-fluorescent target protein bound to a molecular carrier (microtubule) with a length of microscale was unable to pass through the hydrogel structure, while red-fluorescent non-target biomolecules freely penetrated the membrane, resulting in continuous and selective separation of target proteins from protein mixtures. This protein filtration and purification method can be extended to utilize other sieving structures, such as self-assembled nanoparticle clusters [41] or commercial nanoporous membranes [64], etc. The size-based mechanical sieving seems to be advantageous in collecting the target protein by continuously concentrating at the local sieving spot. However, the accumulation at the nanopore interfaces can cause problems with high-concentration solutions or long-term operation, which gradually decreases filtration efficiency over time. This clogging issue can be minimized by horizontal filtration (dynamic filtration) where the direction of flow and membrane formation are on the side. Cheng Y. et al. succeeded in reducing this problem by using a horizontal filtration (i.e., tangential flow filtration) inside the microchannel [64].

### 3.2. Deterministic Lateral Displacement (DLD) Array

DLD technology was developed under specific conditions where micropillar arrays interact with laminar flow [67,68,69,70,71,72,73,74,75,76]. This utilizes a phenomenon in which particles are transported differently for each size depending on the diffusivity of particles, fluid flow resistance, and especially laminar flow parameters [69,76]. The DLD separation of spherical particles begins with the creation of an individual streamline along a defined path due to the lateral gap between the pillars and their row-tilted angle. As shown in schematic trajectories of Figure 1B, Civin et al. [65] developed an automated leukocyte separation device using a high-precision, robust plastic-based microfluidic chip designed for automated preparation of human leukocytes (i.e., WBCs) for flow cytometry, without centrifugation or manual handling of samples.

Devendra et al. [77] coupled DLD with external forces (gravity) to induce particle movement by tilting the microfluidic device for size-specific separation of the mixed-particle solution. Because small particles (4.32 μm) have a smaller critical angle than larger particles (15 μm), it was possible to manipulate the migration by controlling the offset angle. When the offset angle was too large (20°) or too small (5°), the moving direction of the two particles of different sizes was the same, but at a critical angle (10°), where small particles can be transported downside while large particles remain upside, then two kinds of particles were separated in different directions. Depending on the size, the transfer in the lateral direction was due to the pillar structure that repeated along the direction in which the flow of the fluid proceeded, so the difference between trajectories gradually increased, allowing complete separation at the end. Li N. et al. [78] also separated platelets, RBCs, and WBCs using a DLD device composed of two stages. At the first one, sample and buffer flow rate was 0.1 μL/min, which separated blood cells and smaller platelets, then they applied a 10-fold faster flow for further separation of WBCs and RBCs at the second stage. In whole blood separation studies, the author added heparin as an anticoagulant to reduce the viscosity change caused by blood clotting during the separation.

The advantage of a DLD device is adjustability by changing the pillar array design, flow speed, and other additional forces according to target particles size and ranges. On the other hand, particles clogging and binding to the channel surface may be more important due to the increased surface area caused by numerous pillar structures. This implies not only particle loss due to non-specific binding, but also reduced separation performance due to changes in particle trajectories. Precoating the microfluidic channel surface with surfactants, such as Tween 20 and pluronic F-127/F-78, or small protein particles, seems to be an effective solution [67].

### 3.3. Inertial Focusing

Inertial particle focusing in a microchannel flow has tremendous potential for lab-on-a-chip applications due to its advantages of high throughput, simplicity, and external field-free and membrane-free operation, allowing continuous multiparticle separation based on the particle size [42,66,79,80,81,82,83,84,85,86,87,88,89,90,91,92,93,94,95,96,97]. The microfluidic channel profiles used in inertial microfluidics are largely divided into four groups: straight, spiral, sinusoidal, and sudden expansion channels.

In planar straight-channel flow, the parabolic velocity profile due to the non-slip boundary condition on the wall generates a shear-gradient-induced inertial lift force (*F*_LS_) that causes microparticles to move toward the channel wall (Magnus effect). The equation to describe the shear-gradient-induced lift force is *F*_LS_ = *ρ_f_U_m_*^2^*a_p_*^3^*f_L_*/*D_h_*, where *ρ_f_* is fluid density, *U_m_* is average fluid velocity, *a* is the particle diameter, and *f_L_* is the dimensionless lift coefficient, which is a function of the Reynolds number and the position in the cross-section of the channel. As the particles move the channel walls, physical blocking by the wall prevents rotation of the particles and forces them to move away from the channel wall, which induces a guiding force called the wall-induced lift force (*F*_LW_) toward the center of the channel. The equation for the wall-induced lift force is *F*_LW_ = *ρ_f_U_m_*^2^*a_p_*^6^*f_L_*/*D_h_*^4^. Inertial movement refers to a phenomenon in which particles move laterally to an equilibrium position under inertial lift perpendicular to the main flow direction. Thus, the dominant inertial lift, combined with forces in two opposite directions, allows the particles to find equilibrium positions, making narrow bands depending on the particle sizes. Previous studies reported critical conditions for achieving inertial focusing: λ = *a_p_*/*D_h_* > 0.07. The net lift force of the particle in microchannel flow was specified by Asmolov et al. [98]:

(1) $$F_{L}=\frac{f_{L}\rho_{f}U_{m}{}^{2}a_{p}{}^{4}}{D_{h}{}^{2}}$$

When the shape of the microchannel in the longitudinal direction is curved, a secondary flow occurs due to the centrifugal effect and the pressure gradient between the inner and outer walls, which is superposed on the primary flow. This secondary flow is expected to appear as a pair of lateral flows, which are called Dean vortices. The Dean flow promotes the separation effect by applying a Dean drag force (*F*_D_) to the particles to create different equilibrium positions depending on the size or shape of the object. The intensity of the Dean flow can be expressed by a dimensionless number called the Dean number (De = Re(*D_h_*/2*R*)^0.5^), which is determined by the Reynolds number (Re) and the radius of curvature of the channel (*R*).

An additional force used in inertial microfluidics, in addition to the curved design of the microchannel, is the elastic force. This elastic force is present in non-Newtonian viscoelastic fluids, where the elasticity of the fluid induces an additional elastic force (*F*_E_) to the migrating particle. Flow-induced elastic forces generate particle movement and provide precise positioning across the channel cross-section without the need for complex microchannel design. To this end, biocompatible additives, such as polyethylene oxide (PEO) and polyvinylpyrrolidone (PVP), are dissolved to a general aqueous solution to increase the viscosity and induce viscoelastic properties. In summary, straight channels use inertial lift force as a driving force to separate particles, with an additional option to apply an elastic force by adding viscoelasticity to common Newtonian fluids. If the profile of the channel is curved, such as a spiral or sinusoidal, or is designed to repeat sudden expansion and contraction, Dean drag is additionally formed along with inertial lift, enabling more precise particle manipulation. In this case, it is also possible to add viscoelasticity to the fluid by adding an additive that controls viscosity, such as PEO and PVP.

For example, particle separation can be precisely manipulated by designing the microchannel as a curved line, thus creates two counter-rotating flows called Dean vortices in the upper and lower halves of the channel cross-section. [99] Similar to this, Kuntaegowdanahalli et al. [88] also developed inertial microfluidics for continuous particle separation in spiral microchannels. The competition between net lift and Dean drag creates a new balance that moves the particle to its equilibrium position. Particle separation was observed using 10, 15, and 20 μm diameter particles labeled with DAPI, FITC, and TRITC fluorescence to visualize the results of the separation performance. As a result, particles of each size were extracted into three independently separated streams, where the mixture was separated using a spiral microchannel with 130 μm height at flow rate of ~3 mL/min, *D*_e_ = 14.4. Furthermore, the author applied the spiral device for separation of a cell mixture of two sizes with equal cell concentrations (500,000 × 2 cells/mL). The bigger SH-SY5Y cells (~15 μm) and the smaller C6 glioma cells (~8 μm) were split into different outlets with >80% efficiency and >90% cell-recovery rate.

More recently, Xiang N. and Ni Z. [66] developed an electricity-free, hand-held inertial microfluidic sorter for size-based cell separation, which operates by only a single click to release a compressed spring. Using the cell sorter, as shown in Figure 2C(i,ii), the author first demonstrated separation of 7 μm and 20 μm particles to figure out the optimal flow rates. Then, blood samples spiked with stained tumor cells and sheath fluid were injected into the spiral channels at various flow rates. As shown in Figure 2C(iii), the two cells at the outlet showed various distributions according to the applied pressure. The optimal separation efficiency was obtained when the sample and sheath flow rates were 0.2 mL/min and 1.2 mL/min, respectively, where tumor cells were concentrated near the inner channel wall, while blood cells aggregated near the outer channel wall. Because conventional batch-top cell-sorting systems are often bulky and require expensive equipment and facilities, this compact, portable microfluidic sorter appears to be capable of onsite applications that require rapid response, such as sample preparation for infectious diseases.

It is worth discussing the benefits of inertial microfluidic separation that other passive or active methods are difficult to meet. The main advantage is that inertial separation requires high-velocity flow, which spontaneously generates the forces required to separate the particles. This allows for high-throughput separations more than a few mL per minute with a relatively simple setup. Another unique advantage is that the relatively simple device configuration allows hybrid (multiphysics) separations in combination with other separation techniques. In particular, inertial microfluidics enables more sophisticated separations by integrating with active separations, such as dielectrophoresis [40] and magnetophoresis [43], and other passive methods, such as cross-flow filtration [92] and DLD [42]. These multiphysics techniques will be discussed in more detail in the following Section 7.

## 4. Passive Separation Group 2: Gradient-Based Separation

### 4.1. Temperature Gradient

Temperature is a fundamental variable in many fluidic experiments and plays an important role in determining particle transport ability in a fluid as described by the total mass flux equation below:

(2) $$J=-D\nabla c_{i}-cD_{T}\nabla T$$

where *D* is the Brownian diffusion coefficient, *c_i_* is concentration of species *i*, and *D_T_* and *T* are thermal diffusivity and temperature, respectively. There has been active research on thermophoresis, which controls mass transfer and particle motion by forming a microthermal environment (i.e., thermal gradient) in microfluidic devices [100,101,102,103,104,105,106]. This is because temperature is a variable that has a large influence on the kinetic properties of particles and solvents, including viscosity, which provides an advantage in terms of sensitivity compared to standard particle manipulation.

**Figure 3:**
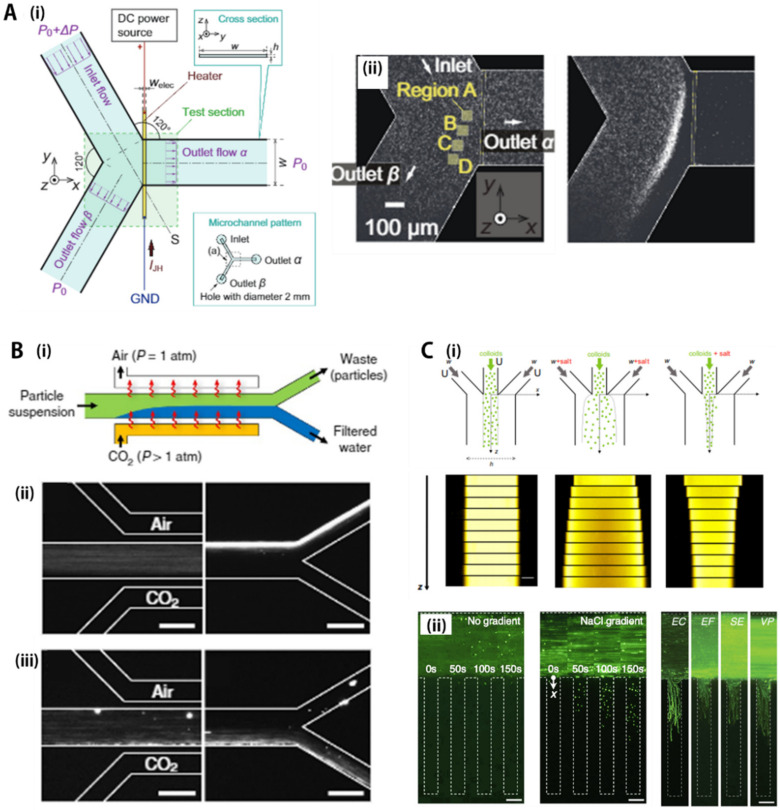
Microfluidic passive-force separation group 2: environmental gradient-based separation. (**A**) (**i**) Illustration to show generation of thermal gradients in microchannel using Joule heating. (**ii**) Separation of particles using thermal gradient, called thermophoresis. Particles separated toward outlet α or β according to their thermophoretic responses. Reprinted with permission from MDPI [101]; (**B**) (**i**) Illustration to describe creation of gas (CO_2_) concentration gradient using air-permeable PDMS microchannel. (**ii**) Separation results using gas concentration gradient. Anionic particles migrated toward high CO_2_ concentration wall, while cation particles were repelled from the high-concentration zone. Reprinted with permission from the Nature Publishing Group [107]; (**C**) (**i**) Particle migration using salt concentration gradient with various operation modes. Reprinted with permission from IOP Publishing Ltd. [108]. (**ii**) Bacterial separation using diffusiophoresis. Negatively charged bacterial cells moved toward high salt concentration channel by salt concentration gradient. Reprinted with permission from the American Chemical Society [109]. All rights reserved.

As shown in Figure 3A(i), a thin film electrode heater was integrated in the Y-shaped microfluidic channel to induce a temperature increase, which maintained the boundary condition of uniform heat flux by applying a DC power source [101]. In addition, a continuous fluid flow using a small pressure difference (0.5 or 1.0 Pa) acts as a heat sink, creating a thermal gradient for thermophoresis of the particles. Figure 3A(ii) shows the movement of particles after heating a thin film electrode. The temperature field reached an almost stationary state in a few seconds, producing a temperature gradient (~0.6 Kμm^−1^) near the electrode. The fluid flow was driven by the pressure difference from the inlet to outlets α and β, then particle flow separation began to be observed over time. In detail, microparticles were unable to enter outlet α, and the area where the particles were concentrated appeared near the upstream thin film electrode; as a result, the particles were flushed out from the stream and directed to outlet α, and a high-concentration suspension was obtained at exit β. At *t* = 250 sec, few particles were present at outlet α, allowing complete particle extraction/separation from fluidic media.

### 4.2. Gas Concentration Gradient

The second term of the total mass flux equation, Equation (2), particle transport by temperature gradient is thermophoresis, and the phenomenon of particle migration by solution concentration gradients is called diffusion-based electrophoresis (i.e., diffusiophoresis), affecting the first term of Equation (2). That is, dissolution of gas molecules into aqueous phases can generate solution concentration gradients that drive phoretic migration of particles [107,110,111,112,113].

As shown in Figure 3B(i), Shin et al. [107] generated the CO_2_ concentration gradient in a gas-permeable microfluidic device that can induce large diffusion potential created by the dissociation of carbonic acid. Due to the gradient, the colloidal particles moved away from or moved toward the gas-liquid interface depending on the surface charge. Specifically, the particle mixture flowed through a straight channel made of PDMS, a gas-permeable material. This channel was located between the air channel and the CO_2_ gas channel, separated by a thin wall, and designed parallel to the main flow channel. The gas penetrated through the wall and dissolved in the sample fluid, causing particle movement perpendicular to the flow direction; however, the transport direction depended on the surface charge of the particle. As shown in Figure 3B(ii,iii), negatively charged particles, which were evenly distributed at the initial state, migrated away from the CO_2_ channel along the flow stream. By contrast, the positively charged particles were filtered using the same principle, and the concentration site moved toward the opposite wall, the CO_2_ channel side. Here, the role of the air channel prevented carbonic acid saturation of the main fluid and played as a sink to maintain a constant CO_2_ concentration gradient along the main flow channel. When all the particles were focused on one side of the channel along the main flow, it was possible to separate and extract the particle-free and the particle-concentrated solutions. Using similar separation schemes, Shin et al. also generated a bacterial-free surface that has great potential for long-term experiments and anti-biofouling applications [113]. The separation principle based on the CO_2_ gas concentration gradient seems to be promising because the gas is abundant and low cost without needing a complex process for inducing the gradient environments, which may be an effective means to generate clean water, especially for resource-limited areas, such as the developing world.

### 4.3. Salt Concentration Gradient

Migration of suspended colloidal particles due to a concentration gradient of solute molecules is basically the same mechanism as the diffusiophoresis performed by the gas concentration gradient. Based on concentration gradients of ionic solutions (e.g., NaCl), many previous studies have verified diffusiophoresis for separation of charged particles in a microfluidic chip in both theoretical and experimental manners [41,114,115,116,117,118,119,120]. Several representative cases are described as schematic diagrams shown in Figure 3C(i) [108]. Without a salt concentration gradient, particles were not dispersed along the flow streamline due to the characteristics of laminar flow and remained unchanged except for random Brownian motion. However, when the colloidal solution was located at the center and flowed with bilateral sheath flows of 10 mM LiCl, the small salt concentration induced significant diffusion of the colloidal band. On the other hand, narrowing (focusing) of the colloidal band was observed when the salt was added to the sheath flows. Interestingly, the bandwidth of the concentrated particles varied depending on ionic concentration and type of background solution, and the effect of focusing or dispersing was reversed depending on the surface charge of the particles.

Diffusiophoresis is simple in principle and requires no additional experimental equipment and facilities, so it can be easily integrated with microfluidic devices using only diffusion-control structures, such as hydrogels, nanochannels, and other nanoporous membranes or structures. For example, Hong et al. [121], fabricated a mixed-scale poly(methyl methacrylate) channel network for single bacterial separation and capture by using diffusiophoresis as a separation driving force. Bacterial cells were introduced along a microchannel containing a low concentration of NaCl (8.55 mM) solution, which was connected to another microchannel filled with M9 medium containing a high concentration of NaCl (1.5 M) to induce diffusiophoresis, resulting in a single bacterial cell that was successfully captured in the microfunnels. Furthermore, Doan et al. [109] demonstrated that even a trace concentration of ionic surfactants, lowered to a single ppm level, can promote bacterial diffusiophoresis by boosting the surface charge of the cells, which were applied for various bacterial strains, such as *E. coli* (EC), *E. faecalis* (EF), *S. enterica* (SE), and *V. parahemolyticus* (VP), as shown in Figure 3C(ii).

## 5. Active Separation Group 1: Non-Contacting Mechanical Forces

### 5.1. Magnetic Force (Magnetophoresis)

Although most bioparticles have no magnetism, the surface of bioparticles can be efficiently functionalized with magnetic properties using various molecular bindings. This functionalization using a strong binding affinity enables selective and sensitive separation of specific bioparticles. In conventional batch bioprocesses, the magnetic force-based separation is one of the most popular methods for extracting specific bioparticles from complex biosample matrices [109] by a serial combination of centrifugation or membrane filtration. However, continuous sample handling, minimization of run-by-run errors, and application to small sample volumes remain challenges. These limitations of conventional approaches have been effectively solved by combining microfluidic technology and magnetophoresis, which continuously separate or extract cells and biomaterials functionalized with magnetism in a microchannel [122,123,124,125,126,127,128,129,130,131]. This means that the degree of freedom in separation is higher because it is possible to give magnetic properties to each particle desired by the end users beyond the limitation of the conventional bioparticle separation, being simple size-based cell separation, resulting in less damage to the samples and excellent biocompatibility.

**Figure 4:**
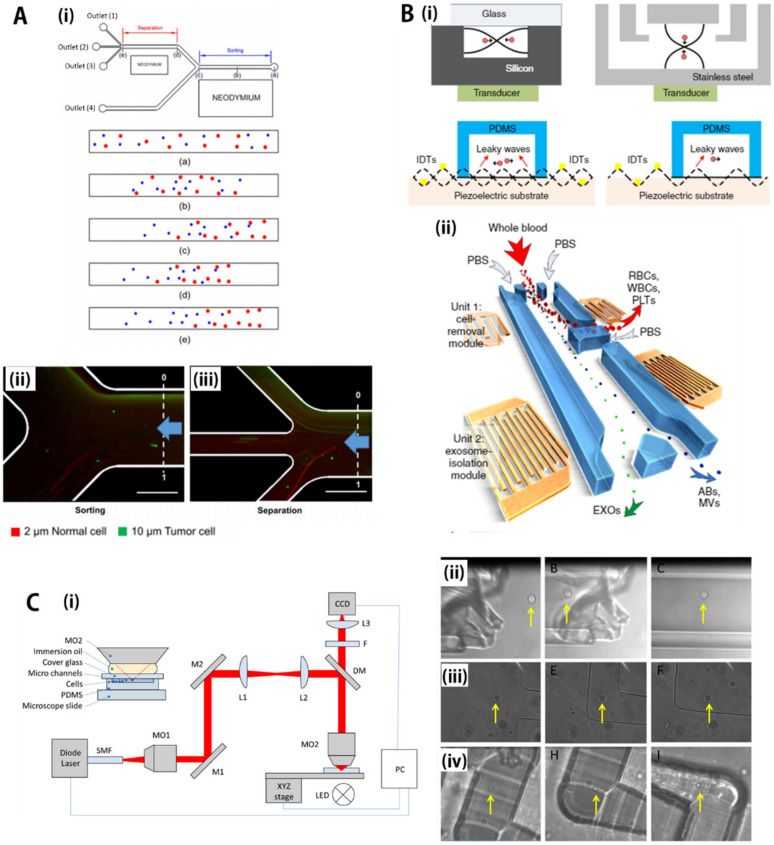
Microfluidic active-force separation group 1: non-contacting, external force-based separation. (**A**) (**i**) Illustration to show sorting and separation using magnetic field (neodymium) acting in microchannels. (**ii**,**iii**) Experimental results showing simultaneous sorting and separation of cells. (**ii**) The upstream magnetism zone was designed for cell sorting and removing tumor cells (**iii**), while the second downstream magnetism zone spectated cells according to their sizes. Reprinted with permission from MDPI [132]; (**B**) (**i**) Schematics to explain various separation setups using transducers that generate acoustic waves. Reprinted with permission from the Nature Publishing Group [133]. (**ii**) Dual-step separations of blood cells and exosomes in blood plasma. The upstream acoustic transducer removed relatively large blood compounds, such as red blood cells, white blood cells, and platelets, then the downstream transducer separated exosomes from other proteins or macrovesicles. Reprinted with permission from the National Academy of Sciences [134]; (**C**) (**i**) Experimental setup for particle separation using optical tweezing with a continuous microflow. (**ii**–**iv**) Microfluidic optical tweezering for manipulation of a single yeast cell (*S. cerevisiae*) using various microfluidic platforms, such as (**ii**) glass micropipette, (**iii**) PDMS chip, and (**iv**) fused silica chip. Reprinted with permission from MDPI [135]. All rights reserved.

For examples, Kye et al. [132], developed dual-neodymium, magnet-based microfluidic separation device, as shown in Figure 4A(i). Specifically, the microfluidic device consisted of two active magnetic zones. One area is for sorting, and the other is for separation. These sorting/separation zones were connected to a common inlet for sample introduction and four separate outlets for extracting microparticles according to their sizes. Smaller microparticles were partially extracted from the first magnetized sorting area to the bottom outlet to reduce the total particulate density of the medium. In the second magnetism zone, the microfluidic device completely separated the microparticles into three outlets according to their size. After optimizing the sorting/separation efficiency using fluorescent microbeads, the authors performed brain cancer cell isolation to demonstrate the applicability of the microfluidic device for cell separation. Sample mixtures with neural stem cells (~2 μm) and glioblastoma cancer cells (~10 μm) were injected into microfluidic separation devices with dual-neodymium magnets. In the first sorting zone, the glioblastoma cancer cells were aligned on the opposite side of the magnet, and neural stem cells were found at the wall on the magnet side. At the second separation zone, most glioblastoma cancer cells were observed at an upper outlet, and neural stem cells were observed at the other outlets as shown in Figure 4A(ii,iii).

### 5.2. Acoustic Force (Accusotophoresis)

Acoustofluidic separation, the fusion of acoustic and microfluidic separation, is based on the interaction of mechanical waves with particles of microfluids. To apply acoustic waves into the microfluidic devices, piezoelectric transducers that can generate mechanical deformation using electrical polarization are prepared near the microfluidic separation channels. Many types of wave generators exist depending on the characteristics of piezoelectric materials, electrode design, and application pattern of electric fields [134,135,136,137,138,139,140,141,142,143]. Basically, an alternating current (AC) signal is applied to the planar piezoelectric transducer that vibrates at the frequency of the AC signal, creating an acoustic wave. According to the vibration modes, there are two representative vibration modes: one is the bulk acoustic wave (BAW), in which the entire transducer vibrates, and the other is the surface acoustic wave (SAW), which vibrates only on the surface of an elastic material. [138,143] As shown in Figure 4B(i) [133], the BAW has relatively simple device architraves with a capability of high-throughput operation. However, microfluidic devices with the BAW are usually made of high-acoustic impedance materials, such as silicon and glass, to induce impedance mismatch between channel materials and fluid medium, which requires a somewhat complicated fabrication process and expertise [144]. On the other hand, the SAW propagates along the surface of a material exhibiting elasticity, with an amplitude that typically decays exponentially with depth into the channel material. Although the throughput is relatively low compared to BAW, the SAW device is suitable for high-precision separation and miniaturization due to the sound velocity mismatch between the substrate and the fluid, creating a significant inertia force and fluid velocity, so it has high compatibility with microfluids [137,143,145].

As shown in Figure 4B(ii), Wu et al. [134] developed a SAW-based acoustofluidic particle manipulation technique that allows the separation of exosomes or other types of extracellular vesicles directly from undiluted whole blood samples in an automated manner by integrating two-step SAW separation on a single chip. First, relatively large blood cells and platelets were removed at the upstream cell-removal module, then the smaller biomolecules still remaining in the plasma were separated into the subgroups of EVs (ABs: apoptotic bodies; EXOs: exosomes; MVs: microvesicles) at the downstream exosome-isolation module. In the upstream cell-removal section, the authors demonstrated the separation of nanoparticles (~110 nm) from a mixture of micro- and nano-sized particles with a yield of more than 99%. In the downstream exosome-separation module, the authors isolated exosomes from the extracellular vesicle mixture with a purity of 98.4%. The integration of two acoustofluidic separation parts to operate continuously on a single chip allowed the separation of exosomes from whole blood with a blood-cell removal rate of more than 99.999%. This automated exosome separation is expected to minimize issues regarding biological risk/contamination by limiting human intervention and to enable large-scale parallel processing with automated processing, enabling convenient integration with downstream exosome analysis devices.

### 5.3. Optical Force (Optophoresis)

Optical manipulation of small particles is referred as an optical tweezer, which uses a highly focused laser beam to trap or move small objects, such as atoms, nanoparticles, and droplets in a manner similar to tweezers [146,147]. The inherent Gaussian distribution of light intensity can generate optical scattering and gradient force, which can provide an attractive or repulsive force, approximately on the order of piconewtons, depending on the difference of the refractive index between particle and background medium. The scattering force pushes particles away from the center of light, while the gradient force attracts particles toward maximum light intensity. Optical tweezers combined with microfluidic technology are used in biological applications to grab and manipulate a single bacterium or cell, such as a sperm or blood cell, or biomolecules, such as DNA [148,149,150,151,152,153].

For example, Wang X. et al. [154] developed a single-cell manipulation method by integrating optical tweezers with microfluidic technologies for sorting and isolating genetically rare cells, such as human embryonic stem cells (hESC), with a high recovery rate and purity. The laminar flow properties of the microfluidic channels can guide the target cells to settle in the desired position for cell separation, and the target cells can be moved precisely in the desired direction with optical tweezers in a non-invasive manner. To this end, the authors devised an image-processing technique to recognize target cells, which differentiated specific cells by recognizing several features, such as cell size and fluorescent labeling. The authors first demonstrated the extraction of yeast cells as target particles from a mixture of microbeads and yeast in DI water to evaluate the performance of the optical cell-sorting system. Microbeads were recognized as non-target particles and flowed through the ROI along the streamline direction without being irradiated with light energy and into the waste channel at the top. In contrast, when yeast cells passed through the ROI, they were detected as target cells, activating optical tweezers, and consequently deviated from the streamline of the laminar flow and moved into the lower collection channel. Similar experiments were applied to isolate pluripotent hESCs from mixtures of multiple cells, which could be important for utilizing hESCs for regenerative medicine and clinical applications. The cell manipulation process was similar to the isolation of yeast cells, except for the use of fluorescence-dependent imaging techniques, since the size of hESCs is very similar to other sizes of the mixture. Another difference from yeast cell sorting is that a higher laser power (1.5 W) is required to sort hESCs. due to a much larger size (i.e., 10–15 μm) than that of yeast cells (i.e., 2–4 μm). The hESCs stained with GFP were driven into the collection channel by an optical tweezer, then flowed into the collection reservoir with the medium flow. After sorting, analysis of the cell populations in each reservoir achieved approximately 90% in both recovery and purity.

As shown in Figure 4C(i), a similar concept was also developed for a single yeast cell (*S. cerevisiae*) separation using various microfluidic platforms made of (ii) glass micropipettes, (iii) PDMS chips, and (iv) fused silica chips [135]. The main advantage of optical force-based bioparticle separation is that particles can be immobilized or manipulated without direct mechanical contact, and the process could be applied to both label-based and label-free methods with relatively precise motion control. However, it could have limitations in terms of small processing volume since each cell needs to be manipulated separately, in addition to a risk of cell damage with excessive exposure to laser light.

## 6. Active Separation Group 2: Contacting Electrical Forces

### 6.1. DC Electric Field (Electrophoresis)

Electrophoresis (EP) is the migration of charged matter, such as ions and particles, suspended in an ionic solution under an externally applied electric field using an electrode set connected to a direct current (DC) source. The external electric field exerts an electrostatic Coulombic force (***F***_E_) on charged particles, which moves the charged particle toward the electrode with opposite charge polarity according to magnitude of the surface charge of the particles. Except for the EP force regarding polarity and charge density, the net EP motion is also affected by the frictional effects and electrophoretic retardation. The friction force (***F***_F_) is a drag on the moving particles due to the viscosity of the surrounding medium, whereas the electrophoretic retardation force (***F***_R_) is caused by the migration of the ion cloud in diffusion layers, known as the Debye layer. The force directions of ***F***_F_ and ***F***_R_ are opposite to the direction of particle movement driven by ***F***_E_. Thus, the net EP force is determined by a force balance among ***F***_E_, ***F***_F_, and ***F***_R_, in the case of a low Reynolds number of fluid flow and moderate electric field strength. The migration velocity (*v*) of a suspended particle is simply proportional to the applied field strength (*E*), as shown in Equation (3):

(3) $$v=\mu_{\mathrm{EP}}\cdot E$$

where *μ*_EP_ is electrophoretic mobility, the measure of charged particle mobility under an electric field. In general, net mobility (*μ*_net_) of a charged particle in a microchannel flow is determined by the combination of *μ*_EP_ with mobilities of bulk flow, such as electro-osmotic flow (*μ*_EOF_) and pressure-driven flow (*μ*_PDF_), which is given in Equation (4):

(4) $$\mu_{net}=\mu_{\mathrm{EP}}+\mu_{\mathrm{EOF}}+\mu_{\mathrm{PDF}}$$

**Figure 5:**
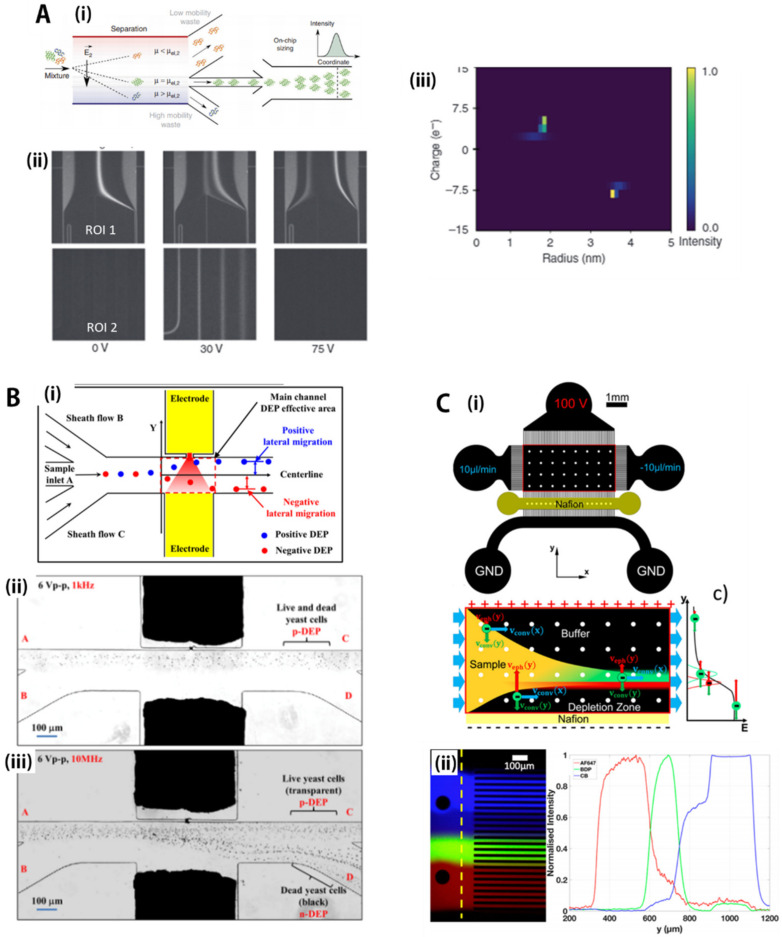
Microfluidic active-force separation group 2: electrical force-based separation. (**A**) (**i**) Illustration to show electrophoresis-based bioparticle separation. (**ii**) Experimental result showing protein separation with electric potential (e.g., 30 V). (**iii**) A two-dimensional separation mapping constructed using microfluidic electrophoresis device with only 3 μL sample over a short time period (7 min). Reprinted with permission from the Nature Publishing Group [155]; (**B**) (**i**) A general scheme for AC dielectrophoresis-based microfluidic device for continuous cell separation. (**ii**), (**iii**) Separation results with improper (i.e., 1 kHz) and optimal AC electric frequency (i.e., 1 × 10^7^ Hz), respectively. Under the optimized electric frequency, the two cell groups showed opposite dielectrophoretic behaviors, showing separation. Reprinted with permission from the American Chemical Society [158]; (**C**) (**i**) Operational principle of particle separation using free-flow ion concentration polarization (ICP) process. The particles initially migrated the cathode due to electro-osmosis, then were separated by the ion depletion region created by the Nafion film, which is an ionically permeable structure. (**ii**) Separation results obtained by the ICP, showing different equilibrium positions according to EP mobilities. Reprinted with permission from the American Chemical Society [159]. All rights reserved.

Many microfluidic EP devices have been developed for accurate motion controls of various small biomolecules, such as nucleic acids, proteins, or metabolites. Most microfluidic separation devices using a DC electric field try to minimize mobilities by bulk flow, making summation of *μ*_EOF_ and *μ*_PDF_ negligible by balancing hydraulic heads and minimizing surface charge density of microchannel walls, called free-flow EP. Saar et al. [155] developed a free-flow EP separation device for analyzing a binary mixture of proteins, such as BSA (*M*_w_= 66 kDa) and human lysozyme (*M*_w_= 15 kDa), that cannot be identified as individual components in native states. Specifically, as shown in Figure 5A(i), the authors separated the protein mixture according to the *μ*_EP_ difference in the separation channel, then analyzed the size distribution of the isolated single type of protein by quantifying the diffusion phenomenon. As shown in Figure 5B(ii), which was the experimental result of applying a voltage range from −75 V to 75 V, an appropriate potential (e.g., 30 V) allowed protein separation, whereas a potential that was too strong (e.g., 75 V) or too weak (e.g., 0 V) failed to direct the proteins into the downstream diffusion sizing section. In particular, the quantitative nature of the EP separation method allowed calculation of the effective charge density and hydrodynamic radius of each constituent fraction because the relationship between migration velocity and applied potential is directly converted to the estimation of *μ*_EP_. The two-dimensional map, as shown in Figure 5A(iii), was constructed using a microfluidic EP device with only 3 μL of sample and a 7 min time period, which is a few orders of magnitude faster than conventional two-dimensional protein gel electrophoresis. In addition to protein analysis, microfluidic EP devices are utilized for various bioparticle manipulations, such as bacterial focusing/immobilization [156], micro-RNA/DNA separation, fingerprinting [45], and drug analysis [157].

### 6.2. AC Electric Field (Dielectrophoresis)

Dielectrophoresis (DEP) is the motion of polarizable objects under an electric field gradient that can induce unbalanced Coulombic forces causing particles to move. Since all particles possess dielectrophoretic activity in an electric field, DEP can be applied universally, unlike electrophoresis that needs specific net charge density of particles. The DEP-based migration of suspended particles is mainly determined by electrical properties of medium and particles, size/shape of particles, and frequency of applied alternating current (AC) electric field. The DEP force (*F*_DEP_) of a homogeneous sphere surrounded by a conducting dielectric medium is given by Equation (5), where *r* is the radius of spherical particle, *ε*_p_ and *ε*_m_ are electrical permeabilities of particle and medium, respectively, and *E* is electric field strength.

(5) FDEP=2πεmr3Re[CM(ω)⋅∇E2(r,ω)]

(6) $$CM=\frac{\varepsilon_{p}-\varepsilon_{m}}{\varepsilon_{p}+2\varepsilon_{m}}$$

The Clausius–Mossotti factor (*CM*) determines migration direction of the particles: positive *CM* (*F*_DEP_ > 0) causes the particle to move to a higher electric field zone, and conversely, negative *CM* (*F*_DEP_ < 0) causes the particle to repel from the strong electric field.

Since biological subjects, such as cells, have dielectric constants, the DEP can be used to manipulate, transport, separate, and sort different types of bioparticles, which is based on differentiation of dielectric and conducting properties of the particles [160,161,162,163,164,165,166,167,168,169]. For example, Figure 5B(i) shows a general scheme for an AC-DEP microfluidic device for continuous cell characterization and separation. The lateral migration can be achieved in the DEP effective area according to the dielectric constant of the particles. First, the bioparticles are prefocused on the centerline by the sheath flows, then enter the DEP active region for separation, where the blue particles migrate to the region of strong electric field strength (positive DEP). By contrast, the red particles move toward a side electrode having relatively weak electric field strength (negative DEP). As shown in Figure 5B(ii,iii), K. Zhao et al. [158] reported microfluidic DEP separation of the live (light dots) and dead (black dots) yeast cells suspended in DI water. When the applied frequency of the AC electric field was 1 kHz (Figure 5B(ii)), which presents *CM* > 0, both live and dead cells experienced positive DEP and were attracted toward the maximum electric field gradient without separation. Under an optimized electric frequency (e.g., 1 × 10^7^ Hz), the two cell groups showed opposite dielectrophoretic behaviors. The live cells *CM* ≈ 0.7 experienced positive DEP behaviors and were pulled toward the strong electric field area (small orifice), then flowed into the cell-collecting channel (outlet C). By contrast, the dead yeast cells that had *CM* ≈ −0.15, experiencing negative DEP, were repelled toward the waste channel (outlet D). Therefore, isolating biological cells of similar size with different dielectric properties exhibited by physiological states of the cells can be achieved by simply adjusting the frequency of the AC electric field in the microfluidic device. In addition, microfluidic DEP techniques are not only utilized for separation of other bioparticles, such as cancer cells from healthy cells and platelets from whole blood, but also applied in biological fields, such as medical diagnostics, drug discovery, cell therapeutics, and particle filtration.

### 6.3. DC Electric Field with Perm-Selective Nanojunctions (Ion Concentration Polarization)

Ion concentration polarization (ICP) is an electrokinetic mass-transport phenomena that occurs when ions selectively penetrate through nanoscale channels or porous membranes, which shows great potential for various applications, such as biomolecule concentration and particle separation, and even for further advanced applications, such as nanofluidic desalination and cell lysis [13,170,171,172,173,174,175,176,177,178]. The fundamental characteristics of the ICP process can be significantly regulated by controlling external parameters, such as applied DC electric potentials, ionic strength of medium, concentration of soluble analytes, and velocity profiles of bulk flows, once the permselectivity of nanofluidic elements is fixed. For particle/solute concentration or separation, ICP phenomena is generally combined with a microfluidic transport mechanism, such as pressure-driven flow (PDF) and/or electro-osmotic flow (EOF), for continuous transport sample analytes, forming micro-/nanofluidic channel networks. The ionic transport phenomena in ICP devices can be described by solving coupled effects among the Nernst–Planck, Navier–Stokes, and Poisson equations, resulting in the flux density of ion species (**J**_i_) as shown in Equation (7):

(7) $$J_{{}_{i}}=D_{i}\nabla c_{i}(t)+\mu_{{}_{i}}z_{i}c_{i}\nabla \phi (t))+c_{i}(t)\cdot u(t)$$

where *D*_i_, *c*_i_, *z*_i_, and *μ*_i_ represent the diffusivity, concentration, ionic valence, and electrophoretic mobility of an ion species i in an electrolyte solution, respectively, and **u** and *φ* mean the fluid velocity and electric potential, respectively. Briefly, the flux density consists of three terms: diffusion, electromigration, and convection. The diffusion term is inversely related to the molecule size and proportional to the diffusivity. The electromigration term is based on charged ions moving in the flow and the electrophoretic mobility of ions. This is also related to the diffusivity defined in the first diffusion term, making coupling effects. The convection term is represented by the sample flow motion, such as PDF and EOF, where the electric potential also affects the EOF of microchannels.

Figure 5C(i) shows a schematic description of the operation principle of free-flow-based ion concentration polarization focusing (ICPF) [159]. A high electric potential is applied to the top reservoir (100 V), and the bottom reservoirs are grounded. The 12-times higher electrical resistance of the array of channels connecting the left and right reservoirs to the separation chamber maintains that most of the current flows only in the direction perpendicular to the flow. As shown in Figure 5C(ii), a sample solution with the negatively charged bioparticles enters into the separation chamber by PDF, then experiences a uniform EOF generated by the perpendicular electric field, which forces the particles to move toward a nearby ion-permselective Nafion film. The moving particle driven by EOF guarantees a force balanced with EP migration because of a precipitous electric field gradient generated by the formation of ion depletion near the Nafion film. Since the EP motion is proportional to the strength of the electric field, it can be enhanced with the electric field gradient, whereas the bulk metric EOF motion has a constant mobility. Thus, the ICP can offer separation effects to bioparticles by making force balances at different positions according to EP mobilities that can be determined by the zeta-potential, sizes, and shapes of particles. As a result, as shown in Figure 5(iii), charged bioparticles, such as fluorescent biomolecules, proteins, or bacterial cells, can be separated by the ICP process where the preconcentration of samples seems to be an additional benefit.

## 7. Discussion: Current Drawbacks and Outlook

### 7.1. Current Issues in Microfluidic-Based Separation Technology

As summarized in Table 1, various microfluidic-based microfluidic technologies have proven their effectiveness in various types of bioparticles and sample metrics over the past decade. However, there remain many challenges to implement these in more diverse and specific fields. The biggest challenge seems to be the throughput limitations resulting from the small sample volumes inherent in microfluidics. In terms of sample preprocessing for biosensing, a small sample volume may not be a big issue, but in terms of an actuator that needs to continuously separate samples, there is still a somewhat large gap between the capacity processed in microchannels (~few μL/min) and the throughput required by the industry (>few L/min). To address this limitation, a separation technique based on fast hydrodynamics, such as inertial microfluidics, seems to be the most suitable, and multilayered, massively parallelized microfluidic separation devices can be a solution [40,179,180]. In particular, the throughput limitation is expected to be effectively overcome as 3D material printers, a multilayer microchannel fabrication technology, begin to be utilized for microfluidic device fabrication.

Another bottleneck in microfluidic separation of bioparticles is sample damage that most active or even several passive separation methods exhibit, which is also related to the throughput issue. This is because excessively high power that damages bioparticles, such as cells, proteins, DNA, etc., is usually applied to biosamples when higher throughput is required when isolating biosamples that are relatively fragile or require recovery, including cells. Most excessive active forces for separation, such as electric fields, acoustic waves, and optical beams, can kill/lyse cells or cause protein and gene modifications. In addition, extremely high flow rate, temperature, and concentration of chemicals used in the passive methods may also induce excessive shear stress, thermal stress (thermal expansion), and osmotic pressure in cells and other bioparticles.

The last issue to be solved in microfluidic separation of bioparticles is the high-resolution separation of nanoscale particles, which is also associated with the aforementioned throughput and compatibility (stability) limitations. In the case of nanoparticles, a larger force gradient or a harsher environment is required than microparticle separation to differentially provide sufficient force to each nanoparticle. In this case, the upper limit of the force magnitude that can be applied is limited by the throughput and sample compatibility. In general, a faster flow is needed for higher throughput, but this may limit the time exposed to separation forces, and for nanoparticles that require a relatively long time compared to microparticles, increasing the throughput may lack sufficient force for separation. Moreover, when excessive power density is applied to compensate for insufficient separation force, compatibility problems, such as damage, dissolution, or deformation of biological particles may occur, so optimization among trade-off parameters is demanded.

### 7.2. Future Research Perspective: Next-Generation Microfluidic Separation Technology

**Figure 6:**
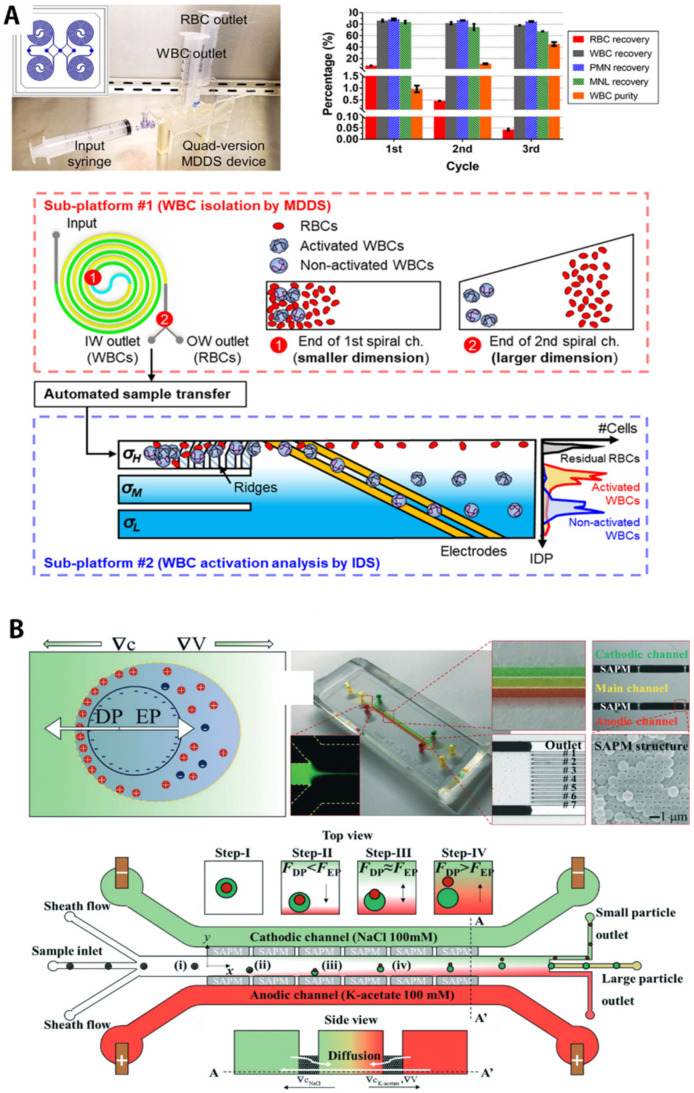
Next-generation microfluidic sample preparation techniques toward high-throughput and high-resolution bioparticle separation with minimal sample damage based on massively parallelized, multiround, and multiphysical separation. (**A**) Multiparallelized and multiround separation for high-throughput isolation of target cells from biosamples with high complexity. Reprinted with permission from the American Chemical Society [40]; (**B**) Multiphysical approach based on combination of passive and active separation mechanisms to minimize sample damage and side effects associated with high electrical field strengths. Reprinted with permission from the Royal Society of Chemistry [41]. All rights reserved.

An effective way to address the aforementioned issues, which may be a future research direction of microfluidic bioparticle separation, is to use massive parallelization of unit separation modules to improve throughput or to use multiple, inline separation forces simultaneously as shown in Figure 6A [40,179,180]. For the parallelization strategy, ultrathin membrane-type 2D microfluidic devices can be utilized to increase separation throughput by height-wise additive manufacturing. In addition, although parallelization is possible by manufacturing a 3D microfluidic device using 3D printing, only a few 3D printing techniques, such as stereo lithography, satisfy the patterning resolution needed for microchannel production (<100 μm), so the advancement of 3D printing technology is expected.

The multiphysical approach also seems to be promising. Passive and active methods can be combined one by one to generate sufficient force to separate nanoparticles while minimizing the use of active forces that can cause sample damage, addressing compatibility issues and allowing for high-throughput separations. For example, Lee et al. [41], presented a combination of electrophoretic and diffusiophoretic migration for nanoparticle separation. Salt concentration gradients, which are a passive generation of a separation force, were created in microchannels where an external DC electric field was applied for accelerating particles using active EP migration (Figure 6B). The potential used for that multiphysics method was only 1–2 V, which is about 10 times smaller than the values typically used for conventional EP isolation (>tens of volts). The small electric force made it possible to separate nanoparticles with high resolution using the synergistic effect of the two forces, which could not be separated when conventional DP or EP were applied alone.

Microfluidic technology for bioparticle separation has been considered to have great potential for biosensing and biomedical analysis applications, enabling continuous, label-free, contamination-free, and biocompatible separations with low consumption of rare biosamples, as well as direct online integration with various biosensors. This separation technique is an efficient method that can directly improve the performance of online downstream biosensors, such as sensitivity, selectivity, and dynamic range, by removing non-target substances present in the biosamples and selectively isolating and concentrating target analytes. However, much effort is still needed to address technical challenges, including system robustness and reproducibility, integration of fluid control modules and sensing modules, and simplified user interfaces, to transform laboratory technology into various biomedical fields. Future work on microfluidic separation technology will focus on developing products and devices for further clinical applications and solving new problems in biology and medicine, in addition to technological improvements for high-throughput or nanoparticle applications. This will allow the microfluidic biological particle separation technology to be applied to more practical applications, which will have a major impact on translational medicine and precision medicine.

## 8. Conclusions

This review aimed to give a comprehensive view of the state-of-art of microfluidic biosample preparation technology for the separation of micro-/nanoscale bioparticles, such as nucleotide acids, proteins, extracellular vesicles, virus, bacteria, blood components, and even human cells. These microfluidic separation platforms have great benefits for many biological research and clinical applications, for not only enhancing selectivity and sensitivity of biosensors but also other clinical tasks, such as cancer therapeutics, treatments of cardiovascular heart disease, extracorporeal hemodialysis, contrast-medium-based bioimaging, drug development, etc. In addition, the various separation principles summarized in this review can be applied to other fields, such as analytical chemistry, development of new functional materials, food analysis, water purification/monitoring, in addition to biological applications.

## Figures and Tables

**Table 1:** Summary of microfluidic separation techniques for separation of various types of bioparticles.

Separation Criteria	Operational Mechanism	Sample Matrix	Target Bioparticles	Throughput/Recovery Ratio/Others	Reference
Hydrodynamic flow-based separation	Sieving/mechanical filtration	PBS buffer with BSA proteins	Aptamer-EGFR conjugate bounded on microtubules	10^5^–10^6^-fold concentration	Kim, M. et al. [62]
	Deterministic lateral displacement (DLD) array	Human blood sample incubated with fluorochrome-conjugated monoclonal antibodies (mAb)	Human leukocytes (WBCs)	200 μL during 18 min, 88% target recovery, 99.985% removal of input erythrocytes, >99% of unbound mAb in 18 min	Civin, C.I. et al. [65]
	Inertial focusing	Diluted blood spiked with pre-stained tumor cells with a concentration of 10^4^ cells/mL	Tumor cells	0.2 mL/min, 78.67% rare tumor cell recovery, >96.04% blood cell removal	Xiang, N. et al. [66]
Micro- environmental gradients-based separation	Temperature gradient (thermophoresis)	Tris-HCl aqueous buffer (pH = 8.0)	0.1 and 1 μm polystyrene particles	V_in_ = 3.5 µm/s	Tsuji, T. et al. [101]
	Gas concentration gradient (diffusiophoresis)	Deionized water	Amine-functionalized polystyrene particles	2 μL/h out of ~2.2 × 10^7^ total particles, only 104 passed during 5 min	Shin, S. et al. [107]
	Salt concentration gradient (diffusiophoresis)	1~100 mM NaCl buffer with 0.1 mM sodium dodecyl sulfate	Gram-positive or -negative, flagellated or nonflagellated bacteria	NA	Doan, V.S. et al. [109]
Non-contacting mechanical force-based separation	Magnetic force (magnetophoresis)	PBS buffer with poly(ethylene oxide)	Glioblastoma cancer cells and neural stem cells	5–13 µL/min, 97 ± 0.8% for 15 μm microparticles	Kye, H.G. et al. [132]
	Acoustic force (accusotophoresis)	Blood or extracellular vesicle mixture solution	Exosomes	4 μL/min, 98.4% purity, > 99.999% blood cell removal rate	Wu, M.X. et al. [134]
	Optical force (optophoresis)	Water, media, or buffer solution	Yeast cells (*S. cerevisiae*) and bacteria (*B. subtilis* and *E. coli*)	V_p_ = 200–300 µm/s	Keloth, A. et al. [135]
Contacting electrical forces-based separation	DC electric field (electrophoresis)	10 mM sodium phosphate buffer at pH 7.4	BSA and human lysozyme proteins	3 μL during 7 min for two-dimensional protein mapping	Saar, K.L. et al. [155]
	AC electric field (dielectrophoresis)	DI water and 0.4–4.8 mM K_2_HPO_4_ solution	Yeast cells (standard lab yeast strain, *Saccharomyces cerevisiae* S288c)	3.75 × 10^−3^ μL/s	Zhao, K. et al. [158]
	DC electric field with permselective nanojunctions (ion concentration polarization)	0.1×PBS buffer and human blood plasma with 3.2% sodium acetate	BODIPY disulfonate	15 μL/min, ~10-fold concentration factor	Papadimitriou, V.A. et al. [159]

## Data Availability

Data is contained within the article.
